# Association of Autoimmune Thyroid Disease with Anti-GAD Antibody ELISA Test Positivity and Risk for Insulin Deficiency in Slowly Progressive Type 1 Diabetes

**DOI:** 10.1155/2018/1847430

**Published:** 2018-07-11

**Authors:** Masahito Katahira, Hidetada Ogata, Takahiro Ito, Tsutomu Miwata, Megumi Goto, Shizuka Nakamura, Hiromi Takashima

**Affiliations:** ^1^Aichi Prefectural University School of Nursing and Health, Nagoya 463-8502, Japan; ^2^Department of Endocrinology and Diabetes, Ichinomiya Municipal Hospital, Ichinomiya 491-8558, Japan; ^3^Department of General Internal Medicine, Matsunami General Hospital, Gifu 501-6062, Japan

## Abstract

The presence of antiglutamic acid decarboxylase antibody (GADA) is required for the diagnosis of slowly progressive type 1 diabetes (SPT1D). We examined the factors influencing GADA determination by radioimmunoassay (GADA-RIA) and by enzyme-linked immunosorbent assay (GADA-ELISA). Sixty patients with SPT1D and 154 patients with type 2 diabetes were examined by both GADA-RIA and GADA-ELISA and for the presence of autoimmune thyroid disease (AITD). We compared the clinical characteristics of these patients based on the positivity or negativity of GADA-RIA and GADA-ELISA, and the existence or nonexistence of AITD. Thirty of 60 (50.0%) GADA-RIA-positive patients were GADA-ELISA negative, whereas none of the 154 GADA-RIA-negative patients were GADA-ELISA positive. Concomitant AITD was significantly less in patients with GADA-RIA and without GADA-ELISA and was significantly more in patients with GADA-RIA and GADA-ELISA. In GADA-RIA-positive patients, there was no significant difference in the GADA-RIA titer among the GADA-ELISA-negative patients with and without AITD, and the GADA-ELISA-positive patients without AITD; whereas the frequency of insulin deficiency was significantly higher in the patients with AITD and/or GADA-ELISA than in those without AITD and GADA-ELISA. Examination of GADA-ELISA and AITD in GADA-RIA-positive patients might be useful in predicting insulin deficiency in these patients.

## 1. Introduction

Type 1 diabetes (T1D) results from *β*-cell destruction that may ultimately lead to a clinical stage in which insulin is required for survival [1]. T1D is divided into three prevalent subtypes: fulminant, acute onset, and slowly progressive [2]. The first two subtypes are abrupt in onset when compared with type 2 diabetes (T2D) and do not necessarily require the presence of islet cell-associated autoantibodies for the diagnosis of T1D [3, 4]. However, slowly progressive T1D (SPT1D) is difficult to diagnose based on the mode of onset and requires the presence of antiglutamic acid decarboxylase antibody (GADA) and/or islet cell antibodies (ICA) [5]. The social health insurance in Japan covers the cost of a GADA test, but not an ICA test, and the presence of GADA is virtually essential for the diagnosis of SPT1D.

In Japan, since December 2015, the assay kit for measuring GADA has changed from radioimmunoassay (RIA) to enzyme-linked immunosorbent assay (ELISA). The ELISA kit was characterized by higher sensitivity and increased specificity for the detection of GADA compared with the RIA kit [6]. Oikawa et al. reported that the positive rate of GADA by ELISA (GADA-ELISA) had a tendency to be higher than that of GADA by RIA (GADA-RIA) in patients with fulminant and/or acute-onset T1D, and that the former had a tendency to be lower than the latter in patients with SPT1D [7]. This discrepancy could have a big effect on the diagnosis of SPT1D.

It was suggested that 15% to 30% of patients with T1D have autoimmune thyroid disease (AITD) [8, 9], and the prevalence of GADA is much higher in patients with AITD than in the general population [10, 11]. The combination of T1D and AITD is called autoimmune polyendocrine syndrome type 3 [12, 13]. The reported mismatch of the results between GADA-RIA and GADA-ELISA tests showed lower GADA-RIA values [6]. Moreover, the logarithm of the GADA-RIA titer was higher in T1D patients with AITD than in those without AITD [14]. From these viewpoints, we speculated that AITD is associated with GADA-ELISA. This study aimed to clarify the differences in the clinical characteristics of patients with SPT1D or T2D based on the positivity or negativity of GADA-RIA and GADA-ELISA, and the existence or nonexistence of AITD.

## 2. Materials and Methods

### 2.1. Subjects

In this study, 60 patients with SPT1D and 154 patients with T2D who visited Ichinomiya Municipal Hospital between December 2015 and February 2016 were enrolled. All patients fulfilled the World Health Organization criteria for diabetes [1], and the patients with SPT1D fulfilled the Japan Diabetes Society criteria for SPT1D [5]. AITD was defined as the presence of Graves' disease, antithyroglobulin antibodies (TgAb), and/or anti-TPO antibodies (TPOAb). Graves' disease was diagnosed according to the guideline proposed by the Japan Thyroid Association [15]. In this study, we defined insulin deficiency based on the fulfillment of the following three criteria: (1) undergoing intensive insulin therapy, (2) not undergoing treatment with glucose-lowering agents that could accelerate insulin secretion (i.e., sulfonylureas, glinides, dipeptidyl peptidase 4 (DPP-4) inhibitors, and glucagon-like peptide 1 (GLP-1) receptor agonists), and (3) fasting C-peptide level of <0.6 ng/mL and/or two-hour postprandial C-peptide level of <1.0 ng/mL. When GADA-RIA was measured more than twice, the maximum titer of GADA-RIA was adopted. When GADA-ELISA was measured more than twice, the value of the first GADA-ELISA measurement was adopted. The study was approved by the ethics committee of the Aichi Prefectural University and Ichinomiya Municipal Hospital.

### 2.2. Measurements

GADA-RIA was determined at sampling using a commercially available RIA kit (GAD-Ab Cosmic, Cosmic, Tokyo, Japan), as described previously [16]. GADA-ELISA was measured using a commercially available kit (RSR Ltd., Cardiff, UK). TgAb and TPOAb were determined by electrochemiluminescence immunoassay (ECLIA) using Roche ECLusys Anti-Tg and Anti-TPO (Roche Diagnostics GmbH, Mannheim, Germany). Reference levels for these parameters were set as follows: GADA-RIA, <1.5 U/mL; GADA-ELISA, <5.0 U/mL; TgAb, <28 IU/mL; and TPOAb, <16 IU/mL. Serum C-peptide level was determined at sampling using a commercially available enzyme immunoassay kit (Kyowa Medex Co. Ltd., Tokyo, Japan). The GADA-RIA, GADA-ELISA, TgAb, TPOAb, and C-peptide tests were outsourced to Health Sciences Research Institute Inc., a commercial laboratory in Japan, and were performed according to the manufacturer's instructions.

### 2.3. Statistical Analysis

Results are presented as medians with interquartile ranges or as numbers. Statistical analysis was performed using PASW Statistics 24.0 (SPSS Inc., an IBM Company, Chicago, IL, USA). Group comparisons of clinical parameters were performed using the Kruskal-Wallis test, Mann–Whitney *U* test, or chi-square test, as appropriate. Multiple comparisons were performed using the Mann–Whitney *U* test with Bonferroni's correction. The correlation between GADA-RIA and GADA-ELISA was analyzed using the Spearman's rank correlation coefficient. The level of statistical significance was defined as *P* < 0.05 or the absolute value of adjusted residual > 1.96.

## 3. Results

### 3.1. Association between GADA-RIA and GADA-ELISA

**Figure 1:**
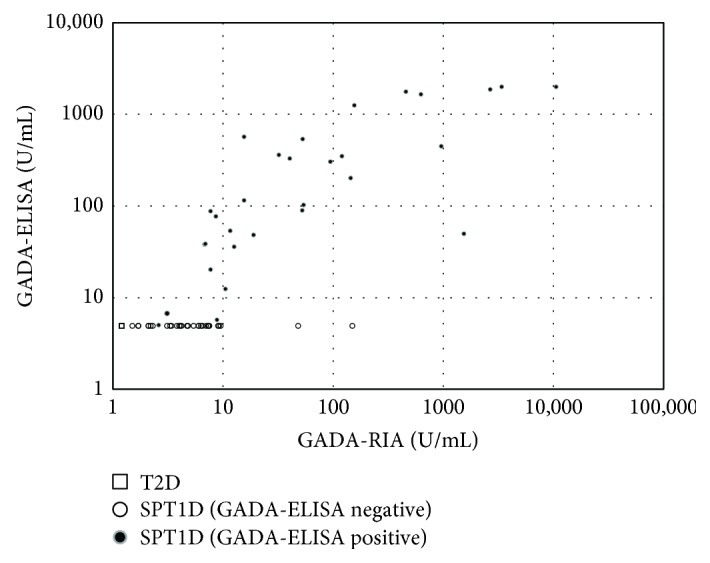
Correlation between GADA-RIA and GADA-ELISA. Dot plots show a significant correlation between GADA-RIA and GADA-ELISA titers in all patients (*ρ* = 0.730, *P* < 0.001), in GADA-RIA-positive patients with SPT1D (*ρ* = 0.778, *P* < 0.001), and in GADA-ELISA-positive patients with SPT1D (*ρ* = 0.819, *P* < 0.001).

In all patients, a significant correlation between GADA-RIA and GADA-ELISA was observed (*ρ* = 0.730, *P* < 0.001) (Figure 1). However, 30 of 60 (50.0%) GADA-RIA-positive patients were GADA-ELISA negative, whereas none of the 154 GADA-RIA-negative patients were GADA-ELISA positive. The positive and negative concordance rate between GADA-RIA and GADA-ELISA was 86.0%. In the GADA-RIA-positive patients (*n* = 60) and GADA-ELISA-positive patients (*n* = 30), the coefficients of correlation (*ρ*) between GADA-RIA and GADA-ELISA were 0.778 (*P* < 0.001) and 0.819 (*P* < 0.001), respectively.

### 3.2. Clinical Characteristics of the Patients Based on GADA-RIA and GADA-ELISA

We divided all patients into three groups based on the positivity or negativity of GADA-RIA and GADA-ELISA: (1) patients without GADA-RIA and GADA-ELISA (group A); (2) those with GADA-RIA and without GADA-ELISA (group B); and (3) those with GADA-RIA and GADA-ELISA (group C). Group A corresponded to patients with T2D, whereas groups B and C corresponded to patients with SPT1D.  Table 1 shows the clinical characteristics of these groups.

The age of onset of diabetes was significantly younger in group C than in group A (49 (38–53) years versus 55 (47–63) years, *P* = 0.028). The titer of GADA-RIA was significantly higher in group C than in group B (36.2 (9.2–152.7) U/mL versus 4.8 (3.2–7.2) U/mL, *P* < 0.001). The interval between GADA-RIA and GADA-ELISA tests was significantly shorter in group B (2.8 (1.7–4.9) years) than in group A and group C (6.6 (2.8–8.5) and 9.0 (4.5–9.7) years, respectively, *P* < 0.001). The frequency of AITD was significantly lower in group B and higher in group C (20.0% versus 66.6%, *P* < 0.001). The frequency of insulin deficiency was significantly lower in group A and higher in group C (0.6% versus 53.3%, *P* < 0.001). The frequency of complete *β*-cell failure (patients with undetected C-peptide level) was significantly lower in group A and higher in group C (0.0% versus 23.3%, *P* < 0.001). The frequency of insulin use was significantly lower in group A and higher in group C (32.5% versus 73.7%, *P* < 0.001). The use of insulin secretagogues (sulfonylureas, glinides, DPP-4 inhibitors, and GLP-1 receptor agonists) and other glucose-lowering agents (biguanides, thiazolidines, *α*-glucosidase inhibitors, and sodium-glucose cotransporter 2 (SGLT2) inhibitors) was significantly more frequent in group A (77.9% and 65.6%, resp.) and less frequent in group C (30.0% and 33.3%, resp.) (*P* < 0.001 and *P* < 0.001, resp.). No significant difference was observed among the three groups with regard to age, gender distribution, body mass index (BMI), glycated hemoglobin (HbA1c), systolic blood pressure, diastolic blood pressure, total cholesterol, high-density lipoprotein (HDL) cholesterol, low-density lipoprotein cholesterol, and non-HDL cholesterol.

### 3.3. Clinical Characteristics of the Patients Based on GADA-RIA, GADA-ELISA, and AITD

We divided groups A, B, and C into two subgroups each based on the presence or absence of AITD: (1) group A without AITD (group A0), (2) group A with AITD (group A1), (3) group B without AITD (group B0), (4) group B with AITD (group B1), (5) group C without AITD (group C0), and (6) group C with AITD (group C1).  Table 2 shows the clinical characteristics of these groups.

The titer of GADA-RIA was significantly higher in group C1 (74.0 (15.5–708.9) U/mL) than in groups B0, B1, and C0 (4.5 (2.3–7.7), 5.7 (4.4–6.4), and 8.3 (6.8–26.7) U/mL, respectively; *P* < 0.001). The titer of GADA-ELISA was significantly higher in group C1 than in group C0 (317.0 (71.1–1354.9) U/mL versus 38.3 (8.1–89.0) U/mL; *P* = 0.005). The frequency of insulin deficiency was significantly lower in groups A0 and A1 (1.0% and 0%, resp.), and was significantly higher in groups B1, C0, and C1 (33.3%, 70.0%, and 45.0%, respectively; *P* < 0.001). The frequency of complete *β*-cell failure (patients with undetected C-peptide level) was significantly lower in group A0 (0.0%) and higher in groups C0 and C1 (30.0% and 20.0%, respectively; *P* < 0.001). The frequency of insulin use was significantly lower in group A0 (32.7%) and higher in groups C0 and C1 (90.0% and 70.0%, respectively; *P* = 0.001). The use of insulin secretagogues was significantly more frequent in group A0 (83.2%) and less frequent in groups B1, C0, and C1 (33.3%, 10.0%, and 40.0%, respectively; *P* < 0.001). The use of other glucose-lowering agents was significantly more frequent in group A0 and less frequent in group C1 (70.3% versus 35.0%; *P* < 0.001). No significant difference was observed among the six groups with regard to age, gender distribution, BMI, HbA1c, age of onset of diabetes, and duration of diabetes.

## 4. Discussion

Although a strong correlation between GADA-RIA and GADA-ELISA was observed in this study, the coefficient of correlation between the two tests in GADA-ELISA-positive patients as well as in GADA-RIA-positive patients was lower than those previously reported in T1D (*r* = 0.925, 0.979) [7, 17]. These previous studies measured the samples by RIA and ELISA simultaneously, whereas this study measured the samples by ELISA after a long interval from the measurement by RIA. In general, GADA values in some patients with SPT1D are considered to decrease over time and finally disappear [5]. This could explain the lower coefficient of correlation between the two tests in this study than that of previous studies.

With regard to the differences in the clinical characteristics between GADA-RIA and GADA-ELISA, in patients with GADA-RIA and GADA-ELISA (group C), the age of diabetes onset was younger, the titer of GADA-RIA was higher, and insulin deficiency and insulin use were higher compared with those without GADA-RIA and GADA-ELISA (group A) and those with GADA-RIA and without GADA-ELISA (group B). These results were in accordance with the previous findings [6]. Because the interval between GADA-RIA and GADA-ELISA tests was shorter in patients with GADA-RIA and without GADA-ELISA (group B) than in those without GADA-RIA and GADA-ELISA (group A) and in those with GADA-RIA and GADA-ELISA (group C), the interval between these tests was less likely to have affected the positive and negative concordance rate between the two tests. It was speculated that the titer of GADA was originally low in group B patients (i.e., positive GADA-RIA and negative GADA-ELISA), which is in accordance with the results of a previous report [6]. On the other hand, this study demonstrated for the first time that the frequency of AITD in GADA-RIA-positive patients was high in those with GADA-ELISA and low in those without GADA-ELISA. This finding suggested that AITD might be associated with the enhancement of GADA-ELISA positivity in GADA-RIA-positive patients. Therefore, we investigated the differences in the clinical characteristics based on the presence of AITD, in addition to GADA-RIA and GADA-ELISA. In group C, the titer of GADA-ELISA was higher in patients with AITD (group C1) than in those without AITD (group C0). This finding indicated the association of AITD with the elevation of the GADA-ELISA titer in GADA-ELISA-positive patients.

In GADA-RIA-positive patients, no significant difference was observed among patients without GADA-ELISA and AITD (group B0), those with AITD and without GADA-ELISA (group B1), and those with GADA-ELISA and without AITD (group C0) with regard to the titer of GADA-RIA, which was high in those with GADA-ELISA and AITD (group C1). However, in GADA-RIA-positive patients, the frequency of insulin deficiency was higher in those with GADA-ELISA and/or AITD compared with those without GADA-ELISA and AITD. It was demonstrated that the presence of AITD contributed to the progression to *β*-cell failure in T1D [18, 19]. The present study suggested that the presence of AITD and/or GADA-ELISA, both of which synergistically elevate the titer of GADA-RIA, might accelerate insulin deficiency in GADA-RIA-positive patients. Moreover, in GADA-RIA-negative patients, the frequency of insulin use was low in those without AITD (group A0). Regardless of its underlying cause, diabetes mellitus is subdivided into three clinical stages: noninsulin requiring, insulin requiring for control, and insulin requiring for survival [1], the third of which corresponds to insulin deficiency. The results in the present study indicated that the percentage of patients in the stage of insulin requiring for control was low in GADA-RIA-negative patients without AITD, suggesting that the presence of AITD might accelerate insulin secretory defects in T2D. Matejková-Behanová et al. demonstrated that the frequency of AITD was high in T2D patients who had low C-peptide levels compared with those who had high C-peptide levels, although they failed to demonstrate any difference in the frequency of AITD between T2D patients treated with hypoglycemic agents and those requiring insulin [20]. Kitano et al. demonstrated in isolated rat pancreatic islets that the serum containing thyroid microsomal autoantibodies significantly suppressed glucose-induced insulin release [21]. Recently, adult-onset autoimmune diabetes with a positive T-cell response, but lacking diabetes-associated autoantibodies has been described [22, 23]. Brooks-Worrell et al. demonstrated that a significantly lower response for stimulated C-peptide was observed in T2D patients with a positive T-cell response compared with those with a negative T-cell response [23]. GADA-RIA-negative patients with AITD in the present study might include patients with a positive T-cell response.

There were several limitations in the present study. First, the small sample from a single institution may raise concerns about generalizing the data, thereby, warranting further investigation. Second, because of the nature of a cross-sectional study design, the causal relationship of the GADA-RIA and GADA-ELISA values with the future progression to an insulin-deficient state remains unknown. To clarify this point, a longitudinal prospective follow-up study will be required in the future. Third, we could not investigate other islet cell-associated autoantibodies such as autoantibodies to insulinoma-associated antigen (IA-2A), insulin (IAA), islet cells (ICA), and zinc transporter 8 (ZnT8A). In general, ICA, IAA, IA-2A, and ZnT8A were more frequent in childhood-onset than adult-onset T1D [24–26]. Sabbah et al. demonstrated that adult patients with T1D had a decreased frequency of multiple autoantibodies compared with childhood patients with T1D [24]. GADA is by far the most common autoantibody in adult-onset diabetes [27]. However, Kawasaki et al. demonstrated that the prevalence of IAA, IA-2A, and ZnT8 in diabetic patients with GADA-RIA is higher than that in T2D patients and that the determination of IAA, IA-2A, and ZnT8 improves the prediction of a future insulin insufficiency in adult-onset autoimmune diabetes [28]. In the present study, patients with insulin deficiency might have had ICA, IAA, IA-2A, or ZnT8A. Fourth, it remains unknown whether the presence of AITD can directly damage *β*-cells. Previous studies demonstrated that diabetic patients with GADA, especially those with a high GADA titer, had a higher frequency of thyroid-related autoantibodies compared with those without GADA [29–31]. In the present study, no significant difference was observed between GADA-RIA-positive and GADA-ELISA-negative patients without AITD (group B0) and those with AITD (group B1) with regard to complete *β*-cell failure. High GADA titer and/or multiple islet cell-associated autoantibodies might cause *β*-cell failure and the positivity of thyroid-related autoantibodies, both of which might not have a direct relationship. Otherwise, the effect of thyroid-related autoantibodies on *β*-cell failure might be milder compared with that of other islet cell-associated autoantibodies.

## 5. Conclusion

This study demonstrated for the first time that the presence of AITD in GADA-RIA-positive patients was associated with the elevation of the GADA-ELISA titer, which probably contributed to the enhancement of GADA-ELISA positivity. The presence of AITD might be associated with insulin secretory defects, not only in SPT1D, but also in T2D. We recommend that in GADA-RIA-positive patients, the presence of AITD and GADA-ELISA positivity should be examined because they might contribute to the progression to *β*-cell failure in these patients.

## Figures and Tables

**Table 1:** Clinical characteristics of the patients based on GADA-RIA and GADA-ELISA.

	T2D	SPT1D		*P* value
	Group A (*n* = 154)	Group B (*n* = 30)	Group C (*n* = 30)	
GADA-RIA	(−)	(+)	(+)	
GADA-ELISA	(−)	(−)	(+)	
Age (years)	68 (61–75)	69 (60–75)	64 (51–69)	0.081
Gender (female/male)	70/84	8/22	17/13	0.057
BMI (kg/m^2^)	23.8 (21.7–27.1)	23.1 (19.3–26.9)	23.9 (21.9–27.0)	0.440
HbA1c (%)	7.0 (6.6-7.6)	6.8 (6.5–7.6)	7.4 (6.7-8.4)	0.158
Age of onset of diabetes (years)	55 (47–63)^∗^	54 (46–65)	49 (38–53)^∗^	0.028
Duration of diabetes (years)	11.1 (5.5–16.6)	8.8 (5.6–16.0)	12.3 (8.9–19.8)	0.389
Interval between GADA-RIA and GADA-ELISA tests (years)	6.6 (2.8–8.5)^†^	2.8 (1.7–4.9)^†‡^	9.0 (4.5–9.7)^‡^	<0.001
Titer of GADA-RIA (U/mL)	—	4.8 (3.2–7.2)	36.2 (9.2–152.7)	<0.001
Titer of GADA-ELISA (U/mL)	—	—	108.8 (40.9–515.0)	—
Presence of AITD	53 (34.4)	6 (20.0)^§^	20 (66.7)^§^	<0.001
Insulin deficiency	1 (0.6)^§^	3 (10.0)	16 (53.3)^§^	<0.001
C-peptide undetected	0 (0.0)^§^	1 (3.3)	7 (23.3)^§^	<0.001
Systolic BP (mmHg)	138 (126–151)	139 (126–146)	144 (130–153)	0.670
Diastolic BP (mmHg)	76 (70–84)	80 (67–86)	76 (68–90)	0.848
Total cholesterol (mg/dL)	186 (164–210)	182 (165–215)	192 (165–216)	0.865
HDL cholesterol (mg/dL)	46 (40–55)	50 (41–59)	51 (42–61)	0.164
LDL cholesterol (mg/dL)	108 (89–128)	107 (86–126)	104 (90–127)	0.944
Non-HDL cholesterol (mg/dL)	133 (117–160)	133 (115–165)	136 (115–149)	0.870
Glucose-lowering agents				
Insulin	50 (32.5)^§^	12 (40.0)	23 (76.7)^§^	<0.001
Insulin secretagogues	120 (77.9)^§^	21 (70.0)	9 (30.0)^§^	<0.001
Others	101 (65.6)^§^	16 (53.3)	10 (33.3)^§^	<0.001

Unless noted otherwise, data are shown as median (interquartile range), or number (%). ^∗^*P* < 0.05, group A versus group C; ^†^*P* < 0.001, group A versus group B; ^‡^*P* < 0.001, group B versus group C; ^§^Absolute value of adjusted residual > 1.96. Insulin secretagogues include sulfonylureas, glinides, DPP-4 inhibitors, and GLP-1 receptor agonists. Others include biguanides, thiazolidines, *α*-glucosidase inhibitors, and SGLT2 inhibitors. T2D, type 2 diabetes; SPT1D, slowly progressive type 1 diabetes; GADA, anti-glutamic acid decarboxylase antibody; ELISA, enzyme-linked immunosorbent assay; BMI, body mass index; RIA, radioimmunoassay; AITD, autoimmune thyroid disease; BP, blood pressure; HDL, high-density lipoprotein; LDL, low-density lipoprotein.

**Table 2:** Clinical characteristics of the patients based on GADA-RIA, GADA-ELISA, and AITD.

Group	T2D		SPT1D				*P* value
	A0	A1	B0	B1	C0	C1	
GADA-RIA	(−)	(−)	(+)	(+)	(+)	(+)	
GADA-ELISA	(−)	(−)	(−)	(−)	(+)	(+)	
AITD	(−)	(+)	(−)	(+)	(−)	(+)	
*n*	101	53	24	6	10	20	
Age (years)	68 (60–75)	68 (64–75)	69 (61–75)	69 (61–72)	60 (50–68)	66 (53–69)	0.359
Gender (female/male)	41/60	29/24	6/18	2/4	4/6	13/7	0.066
BMI (kg/m^2^)	23.8 (22.0–27.4)	23.8 (21.4–26.2)	23.1 (19.3–25.7)	24.4 (20.2–27.9)	23.8 (23.1–26.9)	24.0 (21.1–27.1)	0.726
HbA1c (%)	7.2 (6.7–7.6)	6.9 (6.5–7.6)	6.8 (6.4–7.5)	7.2 (6.6–9.2)	7.3 (6.8–8.2)	7.5 (6.8–8.3)	0.144
Age of onset of diabetes (years)	55 (44–61)	55 (48–65)	54 (44–66)	57 (53–63)	44 (34–53)	49 (43–54)	0.164
Duration of diabetes (years)	11.9 (6.0–17.4)	9.8 (4.9–16.0)	9.3 (5.3–17.9)	8.1 (7.0–9.3	11.4 (9.5–19.6)	12.5 (8.8–19.7)	0.677
Titer of GADA-RIA (U/mL)	—	—	4.5^∗^ (2.3–7.7)	5.7^†^ (4.4–6.4)	8.3^‡^ (6.8–26.7)	74.0^∗^ †‡ (15.5–708.9)	<0.001
Titer of GADA-ELISA (U/mL)	—	—	—	—	38.3 (8.1–89.0)	317.0 (71.1–1354.9)	0.005
Insulin deficiency	1 (1.0)^§^	0 (0.0)^§^	1 (4.2)	2 (33.3)^§^	7 (70.0)^§^	9 (45.0)^§^	<0.001
C-peptide undetected	0 (0.0)^§^	0 (0.0)	1 (4.2)	0 (0.0)	3 (30.0)^§^	4 (20.0)^§^	<0.001
Glucose-lowering agents							
Insulin	33 (32.7)^§^	17 (32.1)	10 (41.7)	2 (33.3)	9 (90.0)^§^	14 (70.0)^§^	0.001
Insulin secretagogues	84 (83.2)^§^	17 (32.1)	19 (79.2)	2 (33.3)^§^	1 (10.0)^§^	8 (40.0)^§^	<0.001
Others (−/+)	71 (70.3)^§^	30 (56.6)	11 (45.8)	5 (83.3)	3 (30.0)	7 (35.0)^§^	0.005

Unless noted otherwise, data are shown as median (interquartile range), or number (%). ^∗^*P* < 0.01, group B0 versus group C1; ^†^*P* < 0.001, group B1 versus group C1; ^‡^*P* < 0.001, group C0 versus group C1; ^§^Absolute value of adjusted residual > 1.96. Insulin secretagogues include sulfonylureas, glinides, DPP-4 inhibitors, and GLP-1 receptor agonists. Others include biguanides, thiazolidines, *α*-glucosidase inhibitors, and SGLT2 inhibitors. T2D, type 2 diabetes; SPT1D, slowly progressive type 1 diabetes; GADA, anti-glutamic acid decarboxylase antibody; ELISA, enzyme-linked immunosorbent assay; BMI, body mass index; RIA, radioimmunoassay; AITD, autoimmune thyroid disease.

## Data Availability

The data used to support the findings of this study are available from the corresponding author upon request.
